# DNA Methylation in *Anopheles albimanus* Modulates the Midgut Immune Response Against *Plasmodium berghei*

**DOI:** 10.3389/fimmu.2019.03025

**Published:** 2020-01-14

**Authors:** Fabiola Claudio-Piedras, Benito Recio-Tótoro, Renaud Condé, Juan M. Hernández-Tablas, Gerardo Hurtado-Sil, Humberto Lanz-Mendoza

**Affiliations:** Centro de Investigaciones Sobre Enfermedades Infecciosas, Instituto Nacional de Salud Pública, Cuernavaca, Mexico

**Keywords:** DNA methylation, resistance to *Plasmodium*, *Anopheles*, immune response, epigenetic regulation

## Abstract

Epigenetic mechanisms such as DNA methylation and histone post-translational modifications are fundamental for the phenotypic plasticity of insects during their interaction with the environment. In response to environmental cues, the methylation pattern in DNA is dynamically remodeled to achieve an epigenetic control of gene expression. DNA methylation is the focus of study in insects for its evolutionarily conserved character; however, there is scant knowledge about the epigenetic regulation in vector mosquitoes, especially during their infection by parasites. The aim of the present study was to evaluate the participation of DNA methylation in the immune response of *Anopheles albimanus* to a *Plasmodium* infection. For this, we first investigated the presence of a fully functional DNA methylation system in *A. albimanus* by assessing its potential role in larval development. Subsequently, we evaluated the transcriptional response to *Plasmodium berghei* of two mosquito phenotypes with different degrees of susceptibility to the parasite, in a scenario where their global DNA methylation had been pharmacologically inhibited. Our study revealed that *A. albimanus* has a functional DNA methylation system that is essential to larval viability, and that is also responsive to feeding and parasite challenges. The pharmacological erasure of the methylome with azacytidine or decitabine abolished the divergent responses of both mosquito phenotypes, leading to a transcriptionally similar response upon parasite challenge. This response was more specific, and the infection load in both phenotypes was lowered. Our findings suggest that DNA methylation may constitute a key factor in vector competence, and a promising target for preventing malaria transmission.

## Introduction

Nucleic acid methylation is the most ancient and conserved epigenetic mechanism (1–4). The methylation of cytosines is a chemical modification of nucleic acids that involves the covalent addition of methyl groups to the 5-carbon of the cytosine (5, 6). This reaction is catalyzed by a family of conserved enzymes called methyltransferases, which place the methyl group on cytosines that lie in the major groove of double-stranded DNA (7–9). DNA methyltransferase 2 (DNMT2) is the most conserved methyltransferase, and it is the only present in dipterans capable of methylating DNA as well as RNA (10–12). Unlike DNMT1 which methylates cytosines in the CpG sites, DNMT2 methylates out of any particular context (6, 10, 11). The 5-methyl-cytosine (5mC) is a stable epigenetic mark that adds information to the genetic code, this mark is dynamic and changes occur in response to environmental stimuli. It does not interfere with base pairing but, depending on the degree of methylation and its context, it can promote or inhibit strand separation (5, 6, 13). The most widespread function of 5mC is to enhance or hinder the binding of transcriptional factors or regulatory proteins that, in conjunction with other epigenetic modifications, modulate the expression of genes through the structure and stability of the chromatin (14, 15). Additionally, the 5mC is the substrate for Ten-eleven Translocation (TET) dioxygenases, which have a key role in the epigenetic dynamics of DNA methylation during the generation and maintenance of phenotypic diversity (16, 17). The family of TET enzymes is formed by TET1, TET2, and TET3 (17). TET enzymes catalyze the oxidative demethylation of 5mC to form more oxidized intermediates of cytosine that can be converted back to unmodified cytosines (17).

In the dipteran model *Drosophila*, the amount of global 5mC is about as low as 0.1–0.6% of all cytosines (18–20) and the null mutation of the *dnmt2* gene showed no apparent anomalies in development (21). This low levels of DNA methylation present in dipterans has caused it to been regarded as having a subtle function or even a lack thereof (22, 23). Despite this, the biological significance of this epigenetic mark in *Drosophila* has been established in the silencing of retrotransposon transcription, maintenance of telomere integrity (20), and as a requirement for a normal lifespan (21). Furthermore, DNMT2 is essential for efficient immune antiviral responses (24) and the protection of RNA under heat shock and oxidative stress in *Drosophila* (25). Other insects like bees, wasps, and sawflies have more complex methylation system comprising three enzymes; DNMT1, DNMT2, and DNMT3 (23). For example, *Apis mellifera* have ~1.4% of their total genomic cytosines methylated (4) and their methylation system has been studied in relation to transcriptional activity and phenotypic plasticity (1, 4, 26, 27). In other insects such as horned beetles, *Tribolium castaneum*, and *Galleria mellonella*, DNA methylation has been shown to play a role in the generation of different phenotypes, changes in behavioral and nutritional plasticity, as well as in stress responses induced by heat or infections (28–30). In mosquitoes, it is not well-understood whether DNA methylation plays an essential role in their development, nutrition, or immune response (31–35).

*Anopheles albimanus* is one of the principal malaria vectors in Central America and its surroundings from Florida and Texas in the north, to Peru in the south (36, 37). Mosquito infection by *Plasmodium* depends on the parasite development into invasive ookinetes that colonize and form oocysts in the midgut (38, 39). The most significant reduction in parasite numbers occurs in the midgut, which displays a fast immune response (36, 40, 41). Since the innate immune response constitutes an insect's primary defense mechanism against infections, several mosquito immune effectors are related to different degrees of resistance to *Plasmodium* (41–44). *Anopheles albimanus* has two naturally occurring phenotypes with differences in their *P. vivax* infection susceptibility (45). These phenotypes are distinguished on larvae and pupae by the presence or absence of a morphological marker denominated *stripe*. The *white stripe* phenotype shows a layer of white pigment visible below the cuticle of the back of the abdomen and the thorax. The *white stripe* mosquitoes (White, W) have been shown to be more susceptible to *P. vivax* infections than the non-striped mosquitoes (Brown, B) (45). This phenomenon has not been characterized at the molecular or functional level. The natural occurring of phenotypes in *A. albimanus* may be involved in various physiological processes in the mosquitoes, in which DNA methylation can play an important role. We took advantage of this phenotypic trait to study the influence of DNA methylation in the transcriptional immune response to a parasite challenge in susceptible and resistant mosquitoes.

## Results

### *Anopheles albimanus* Has Functional Genes of the DNA Methylation Machinery Which Are Required for Mosquito Development

**Figure 1:**
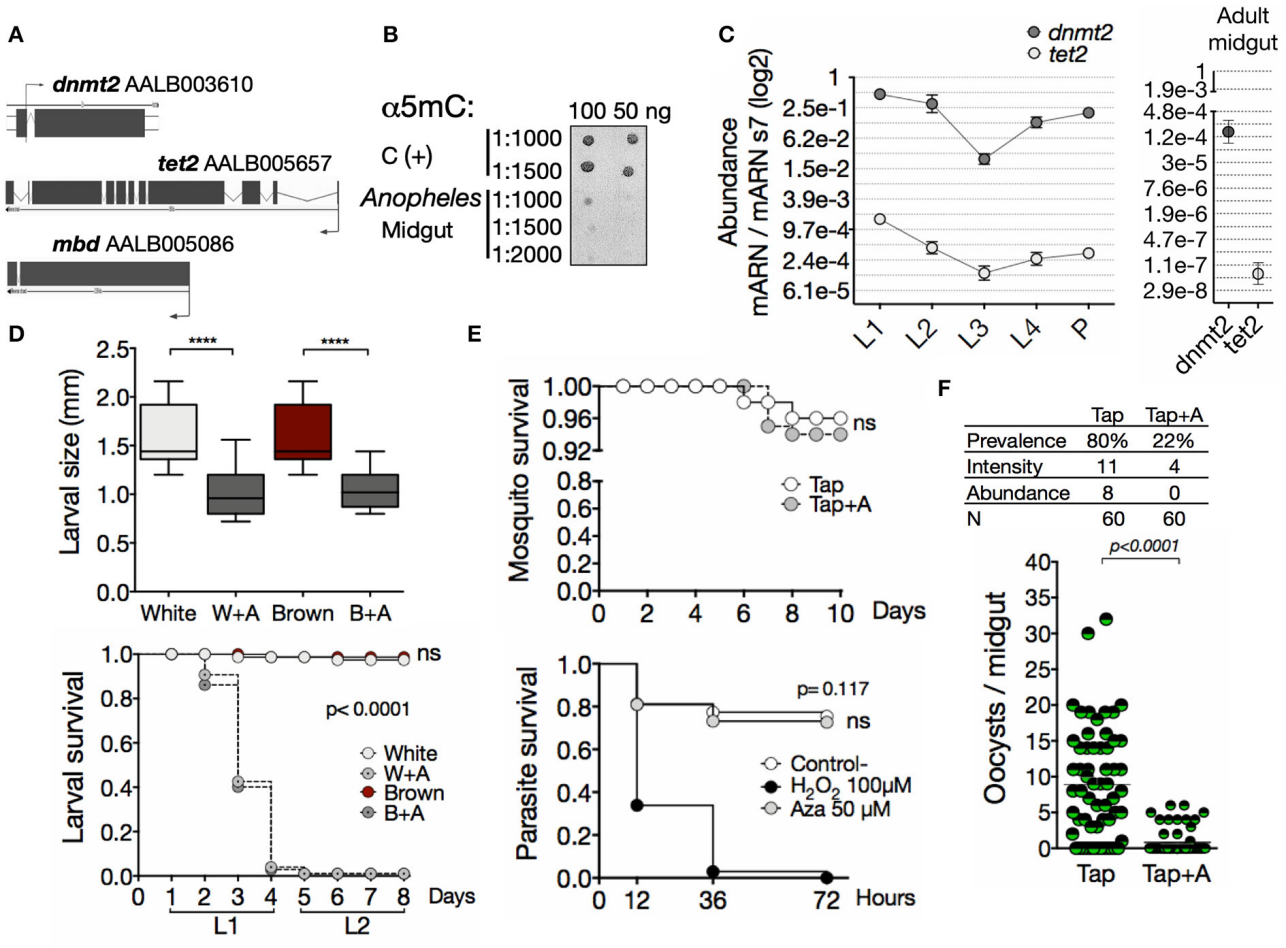
A functional methylation system is involved in the development of *Anopheles albimanus* and their infection by *Plasmodium*. **(A)** *In silico* identification of the basic methylation system. **(B)** Immuno-detection of 5mC in midgut gDNA. C (+), hypermethylated control. 1:1,000, 1:1,500, and 1:2,000 denote α5mC dilutions. **(C)** Expression of *dnmt2* and *tet2* during development and adulthood, determined by qPCR. Mean ± standard error of the mean (SEM) of two independent experiments. **(D)** Larval size 4 days post hatching (two independent experiments with 30 larvae per group) and survival (two independent experiments performed in triplicates) after treatment with azacytidine. *P* values were calculated by Kruskal-Wallis followed by Dunns test and Mantel-Cox test, respectively: ****P < 0.0001. **(E)** Mosquito survival of the parental (Tap) strain and *in vitro* parasite survival following 72 h of 50 μM azacytidine treatment. For parasites, PBS was used as negative control and H_2_O_2_ as positive control. Mean ± SEM of three independent experiments analyzed by Mantel-Cox test. **(F)** Parasite infection following azacytidine treatment of the Tap strain. Two independent experiments with 30 mosquitoes for each experiment and analyzed by Mann-Whitney test. Infection parameters were calculated as follows: prevalence, percentage of infected mosquitoes; intensity, average number of oocysts per infected mosquito; abundance, average number of oocysts per total mosquitoes.

To obtain insights regarding if the methylation system is operating in *A. albimanus*, we initially took a bioinformatic approach to look for the DNA sequences coding for the proteins involved in DNA methylation. A search in the *A. albimanus* genome database revealed the presence of genes coding for a *DNA/RNA methyltransferase 2* (*dnmt2*), a demethylase *methylcytosine dioxygenase tet2* (*tet2*) and the regulatory protein, *methyl-CpG-binding domain protein* (*mbd*) (Figure 1A and Figures S1–S3). Hence, the *A. albimanus* genome presents all the components needed for regulating gene expression by nucleic acid methylation. Given that the first organ challenged by *Plasmodium* invasion is the midgut (38), we particularly focused on this organ and determined whether the genomic DNA (gDNA) and RNA in this organ contained 5mC. We found the epigenetic mark in the midgut DNA (Figure 1B; mean pixel intensity: C+ = 44.2 ± 3.2, Midgut = 10.26 ± 2.3) and RNA (Figure S4; 5mC% = 0.73 ± 0.11, after azacytidine treatment 5mC% = 0.48 ± 0.16). Since DNA methylation has essential roles in reproduction and development, orchestrating the phenotypic plasticity of almost all organisms (46), we determined its functionality during mosquito development. First, we evaluated the transcription profile of *dnmt2* and *tet2* during ontogeny. Both genes were transcribed throughout the mosquito development and during adulthood (Figure 1C). Then, the effect of methylation inhibition with azacytidine in the early stages of larval development was evaluated, resulting in diminished size and survival of the larvae (Figure 1D). Together, these data provide strong evidence for the functionality of the methylation system and demonstrate an essential role of nucleic acid methylation during the ontogeny of the mosquito. In contrast, the survival of adult mosquitoes and ookinetes was not affected (Figure 1E). Interestingly, the azacytidine treatment significantly reduced the mosquito infection (Figure 1F), suggesting that nucleic acid methylation plays a key role during this process.

### *Anopheles albimanus* Phenotypes Are Different in gDNA Methylation Status, Transcriptional Profile, and Susceptibility to *Plasmodium*

**Figure 2:**
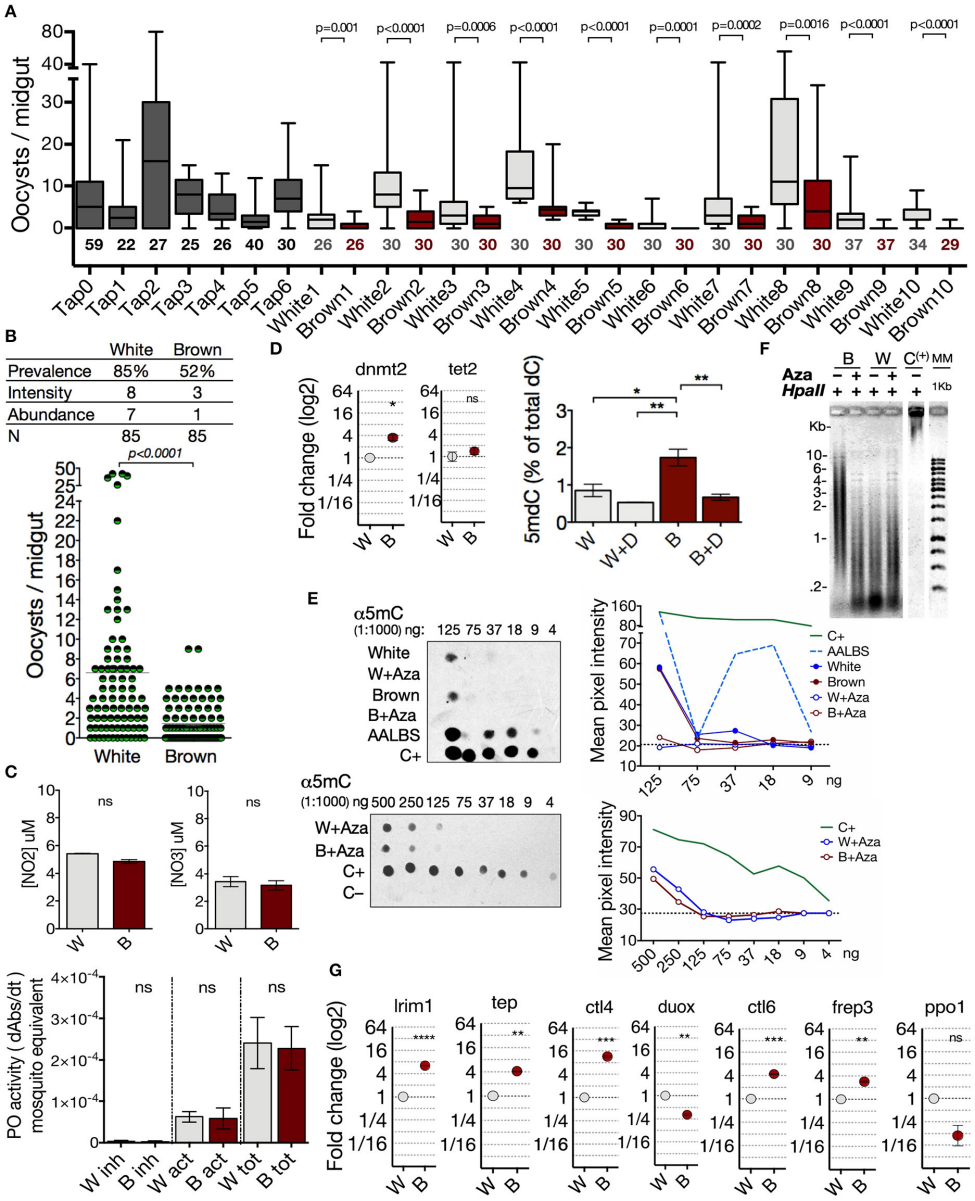
*Anopheles albimanus* phenotypes are different in gDNA methylation status, transcriptional profile and susceptibility to *Plasmodium*. **(A)** Intergenerational follow-up of the infection abundance in 7 generations of the parental Tap strain and 10 generations of the Brown and White derived phenotypes. Numbers below the boxes indicate the number of mosquitoes sampled. Data was analyzed by Mann-Whitney test. **(B)** Infection parameters in the White-susceptible and Brown-resistant phenotypes. Results of three independent experiments analyzed by Mann-Whitney test. **(C)** Measurement of nitric oxide production, and phenoloxidase activity in the White (W) and Brown (B) phenotypes. Inhibited enzyme (inh), active enzyme (act) and total enzyme (tot). Mean ± SEM of three independent experiments analyzed by two-tailed *t* test. **(D)** Basal midgut expression of *dnmt2* and *tet2* in Brown (B) and White (W) mosquitoes obtained by qPCR and the percentage of global 5-methyl-2-deoxycytidine (5mdC) in midgut gDNA of both phenotypes treated with 50 μM of decitabine (W+D and B+D). Determinations were obtained by HPLC (mean ± SEM of three independent experiments, analyzed by ANOVA and Tukey *post-hoc* test). **(E)** Effect of azacytidine on the amount of 5mC in midgut gDNA of the mosquito phenotypes determined by immunodetection. Mean pixel intensity of the dots is represented to the right of the blots. Hypermethylated DNA as positive control (C+), *lambda* phage as negative control (C–). AALBS is the *A. albimanus* LSB-AA695BB cell line. The numbers above the blots represent gDNA dilutions. **(F)** Midgut gDNA digestion with *HpaII* after azacytidine treatment in both phenotypes. (C+) = hypermethylated DNA. **(G)** Basal midgut expression of several immune anti-*Plasmodium* markers in the two phenotypes. Expression determined by qPCR and relative to White mosquito's expression. Means ± SEM of three independent experiments analyzed by two-tailed *t* test. Asterisks represent the *P* value as follows: *P < 0.05, **P < 0.01, ***P < 0.001, ****P < 0.0001.

Epigenetic modifications have been shown to modulate phenotype expression in insects (26–30). Thus, to explore the potential role of the DNA methylation in the phenotypes of *A. albimanus* and their responses against a *Plasmodium* infection, we first characterized the two phenotypes from the parental mosquito Tapachula (Tap) strain at a functional and molecular level. We determined the susceptibility/resistance condition of the White and Brown phenotypes to *P. berghei* infections. For this, female mosquitoes of 5 days post-emergency were fed with 400 ookinetes per μl, and 3 days post-feeding, the oocyst load per midgut was determined by fluorescence microscopy. This experiment was repeated for seven generations for the Tap strain and for 10 successive generations for White and Brown. In each generation of the Tap strain and the derived Brown and White phenotypes, a sample of mosquitoes were taken for the parasite challenges, while the rest were kept for breeding the next generation. As shown, *P. vivax*-susceptible White mosquitoes are also more susceptible to *P. berghei* infections compared to Brown mosquitoes (Figures 2A,B, and Figure S5).

To characterize the phenotypes at a molecular level, we evaluated basal nitric oxide production, phenoloxidase activity, amount of genomic 5mC, and transcriptional profile of several immune markers previously described as the main effectors in the mosquito midgut against *Plasmodium* (36, 40). The basal nitric oxide (NO) production and phenoloxidase (PO) activity were not different between the phenotypes (Figure 2C). The differences in susceptibility observed between phenotypes did not correlate with basal NO production or PO activity, so it is not a feature that conditions the susceptibility/resistance to infection in the phenotypes.

Next, we analyzed the transcriptional profile of *dnmt2* and *tet2*, and the 5mC content in both phenotypes. With a 3.5-fold higher *dnmt2* transcription, Brown mosquitoes had significantly more total gDNA methylation (1.73% ± 0.22) than White mosquitoes (0.85% ± 0.17) (Figure 2D). We found that the amount of the nucleoside 5-methyl-2-deoxycytidine (5mdC) between phenotypes was leveled after the treatment with decitabine; an exclusive DNA–methylation inhibitor (Figure 2D). Furthermore, azacytidine inhibited DNA methylation in both phenotypes, although slightly more efficiently in Brown mosquitoes (Figures 2D–F). After gDNA digestion with the 5mC-sensitive restriction enzyme *HpaII*, Brown mosquitoes showed digestion protection which is lost after azacytidine treatment (Figure 2F), thus corroborating a higher degree of gDNA methylation than in the White mosquitoes.

Then, evaluation was made of the basal transcription levels of *leucine-rich repeat immune protein 1* (*lrim1*), *thioester-containing complement-like protein* (*tep*), *c-type lectin 4* (*ctl4*), *dual oxidase* (*duox*), *c-type lectin 6* (*ctl6*), *fibrinogen-related protein 3* (*frep3*), and *pro-phenoloxidase 1* (*ppo1*). We found several differences between the transcription levels in the phenotypes. Fold change transcript abundance of Brown in relation to White mosquito were as follows: *lrim1* (+6.3), *tep* (+4.1); *ctl4* (+11.6), *ctl6* (+4.4), *frep3* (+2.8) (Figure 2G). In *A. gambiae* and *A. stephensi*, Dual oxidase acts as a modulator that prevents a strong immune response (47, 48). Thus, the lower transcription (3.1-fold) of *duox* in Brown mosquitoes could also contribute to its resistance condition (Figure 2G). No differences were observed in the pro-phenoloxidase 1 (*ppo1*) expression (Figure 2G). These mosquito transcriptional profiles correlate with the susceptibility differences observed between the White (prevalence = 85%, intensity = 8, abundance = 7) and Brown (prevalence = 52%, intensity = 3, abundance = 1) phenotypes (Figure 2B). Our results show significant differences between the phenotypes, which consist of a higher abundance of genomic 5mC, higher transcriptional activity of immune markers in basal conditions, and a lower load of oocysts in the Brown phenotype compared to the White phenotype. Considering the transcriptional differences and amount of 5mC observed between White and Brown mosquitoes, we explored the possibility that these differences would be modulated by gDNA methylation.

### White and Brown Phenotypes Differ in Their Modulation of Immune Genes by Methylation

**Figure 3:**
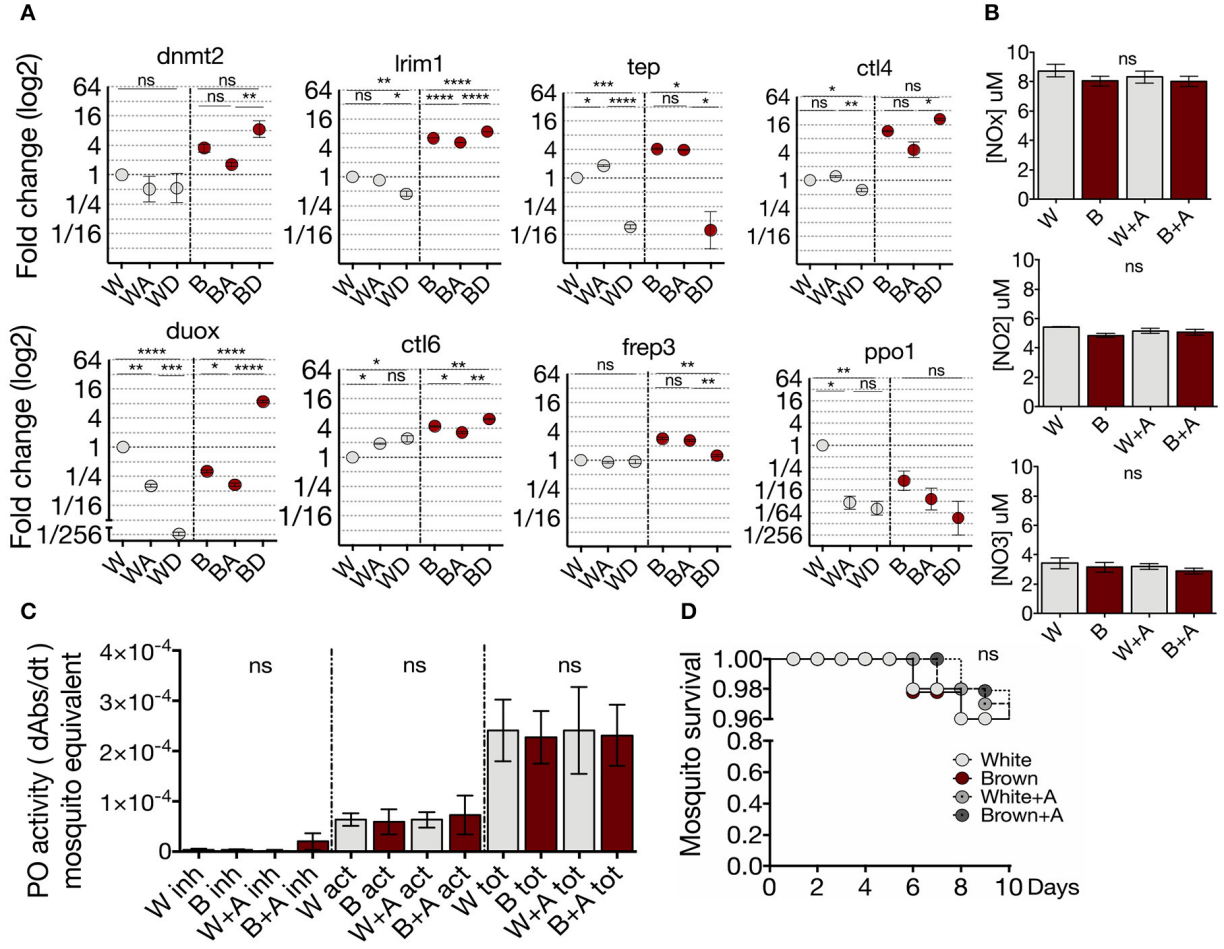
White and Brown phenotypes differ in their regulation of immune genes by methylation. **(A)** Effect of azacytidine and decitabine in immune gene expression of the phenotypes determined by qPCR and relative to White mosquito's expression. Mean ± SEM of three independent experiments analyzed by ANOVA and Tukey *post-hoc* test. **(B)** Effect of the azacytidine treatment on nitric oxide production and **(C)** phenoloxidase activity in White (W) and Brown (B) phenotypes. Inhibited enzyme (inh), active enzyme (act), and total enzyme (tot). Mean ± SEM of three independent experiments analyzed by ANOVA and Tukey *post-hoc* test. **(D)** Brown and White mosquito survival after 72 h of treatment with 50 μM of azacytidine. Mean ± SEM of three independent experiments analyzed by Mantel-Cox test. Asterisks represent the *P* value as follows: *P < 0.05, **P < 0.01, ***P < 0.001, ****P < 0.0001.

To investigate whether transcription of the immune markers selected are modulated by DNA or RNA methylation, White and Brown mosquitoes were treated with the azanucleosides decitabine or azacytidine, and their transcription profiles were determined. The methylation inhibitor decitabine is a deoxycytidine analog that is exclusively incorporated into DNA, while azacytidine incorporates into DNA and RNA (49). Inhibition of methylation by the azanucleosides had phenotype-specific effects on transcription (Figure 3A). This effect may be due to the differential deposition of epigenetic marks between the phenotypes, which can depend on underlying genetic differences (50). Remarkably, decitabine produced greater and in many cases contrary transcriptional effects than azacytidine. It has been shown that a loss of 5mC in RNA causes impairments in post-transcriptional processes (51), whereas the effect of decitabine only involves transcriptional processes. The selective inhibition of DNA methylation affects the abundance of distinct transcripts among the phenotypes (White: *lrim1* and *ctl4*; Brown: *frep3*) (Figure 3A). In White mosquitoes, transcript abundance of *ctl6* and *ppo1* were affected mainly by 5mC inhibition in the DNA (the inhibition of RNA methylation by azacytidine did not aggregate transcriptional effects). There were no changes in transcript expression resulting exclusively from RNA inhibition (by azacytidine but not decitabine). In contrast, both inhibitors affected the expression of some gene transcripts in White mosquitoes (*tep, duox, ctl6*, and *ppo1*) and Brown mosquitoes (*lrim1, duox* and *ctl6*), although with opposite effects (White: *tep*; Brown: *lrim1, duox*, and *ctl6*). In contrast, 5mC inhibition did not have any post-transcriptional effect on NO production (Figure 3B) and phenoloxidase activity (Figure 3C). Moreover, the methylation inhibition treatment did not have any impact on the adult survival of both phenotypes (Figure 3D). Our results show that the inhibition of methylation had phenotype-specific effects on the midgut transcription profile. Furthermore, these data provide evidence that the midgut expression of anti-*Plasmodium* genes is epigenetically modulated by DNA methylation in *A. albimanus*.

### White and Brown Phenotypes Respond Differently to a *Plasmodium* Challenge in Terms of DNA Methylation and Transcription of Immune Genes

**Figure 4:**
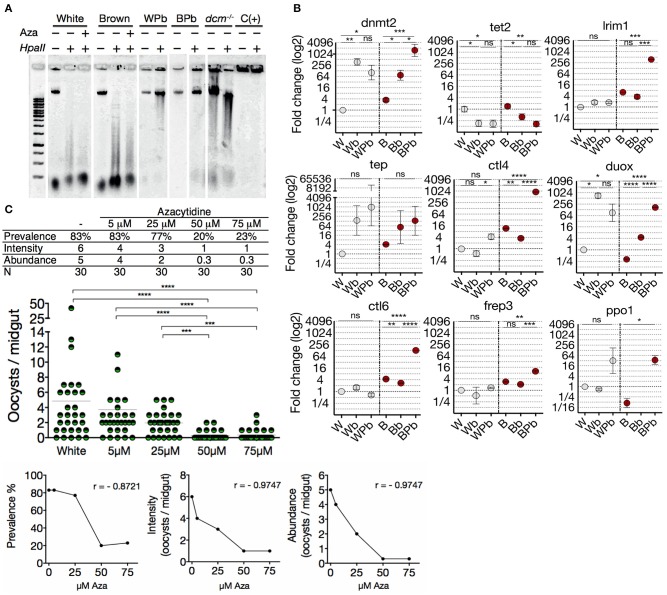
White and Brown phenotypes respond differently to a *Plasmodium* challenge in terms of DNA methylation and transcription of immune genes. **(A)** Midgut gDNA digestion with *HpaII* after *Plasmodium* infection or azacytidine treatment. *Escherichia coli* BL21 *dcm*^−/−^ as a hypomethylated control, hypermethylated DNA as positive control C(+), White mosquitoes challenged with ookinetes (WPb), Brown mosquitoes challenged with ookinetes (BPb). **(B)** Transcriptional response of immune genes in White and Brown mosquitoes after an uninfected blood meal (mock culture; Wb and Bb, respectively) or *Plasmodium berghei*-infected blood meal (WPb and BPb, respectively) determined by qPCR and relative to White mosquito's expression. Mean ± SEM of three independent experiments, analyzed by ANOVA and Tukey *post-hoc* test. **(C)** Effect of different concentrations of azacytidine on the infection of the White-susceptible phenotype and correlation between the concentration of azacytidine treatment and infection prevalence, intensity, and abundance. Data was analyzed by Kruskal-Wallis and Dunn's multiple comparisons test. (*r*) Spearman correlation *p* < 0.0001. Asterisks represent the *P* values as follows: *P < 0.05, **P < 0.01, ***P < 0.001, ****P < 0.0001.

Since oxidative stress, nutritional balance, and microbial challenges trigger epigenetic marking of DNA (52–54), we tested the functionality of the methylation system of both mosquitoes phenotypes. For this we evaluated whether the status of 5mC in midgut gDNA changes in response to an immune challenge with the parasite. Midgut gDNA of *Plasmodium*-challenged mosquitoes of both phenotypes were digested with the restriction enzyme *HpaII*. Midgut gDNA of *Plasmodium*-challenged mosquitoes of both phenotypes showed less sensitivity to *HpaII* digestion (Figure 4A), revealing a higher level of 5mC and a dynamic methylation status that responds to the immune challenge. In agreement with this epigenetic response, clearly induced from the immune challenge, we found that *dnmt2* and *tet2*, as well as most of the anti-*Plasmodium* genes have putative regulatory binding sequences for the nuclear factor-kB (NF-kB) family, the key transcription factors of the immune response (55) (Table S1). Therefore, we evaluated the transcriptional profile of mosquitoes fed with uninfected- and *P. berghei* infected-blood. Upon parasite challenge, Brown mosquitoes showed an increase in *dnmt2* transcription (BPb/Bb: 21.3-fold) and a tendency to decrease *tet2*, while the White mosquitoes did not show significant differences (Figure 4B). This balance is congruent with the gain in the epigenetic mark. Also, blood-feeding induced a substantial increase in *dnmt2* transcription (Wb: 372-fold; Bb 21-fold) and a decrease in *tet2* (Wb: 5.6-fold; Bb: 3.6-fold). Hence, we corroborated that *dnmt2* and *tet2* are responsive to environmental stimuli.

*Anopheles* mosquitoes regulate their transcriptional profile during *Plasmodium* invasion (36). The specificity and quantity of this response can contribute to the resistance to the parasite, therefore to obtain further insights into the susceptibility differences; we evaluated the transcriptional response to the parasite in both mosquito phenotypes. We found that the transcription profile of Brown vs. White mosquitoes during *Plasmodium* invasion was strongly induced and significantly most robust (Figure 4B). Furthermore, the only significantly up-regulated gene in White mosquitoes was *ctl4* (7.7-fold), a lectin-like receptor considered an agonist of *Plasmodium* survival in the midgut (56). In contrast, Brown mosquitoes drastically increased the transcription of *lrim1* (104-fold), *ctl6* (47.6-fold), *frep3* (4.9-fold), and *ppo1* (286-fold) which correlates with its resistance condition despite the increment in *ctl4* (260-fold) and *duox* (37.8-fold). Then, to assess the impact of 5mC on the susceptibility condition, the methylation pattern was altered in the White phenotype with azacytidine 3 days before the ookinete challenge. A strong correlation was found between a lower quantity of oocysts and a higher concentration of azacytidine (Figure 4C). The effect bottomed at 50 μM of azacytidine showing no further decrease in oocyst infection. We then asked if the gDNA methylation status differences between the two phenotypes could be related to the differences observed in *P. berghei* susceptibility. By inhibiting methylation with azacytidine before mosquito exposure to the parasite, a lower prevalence and intensity of infection was exhibited by both the White and Brown mosquitoes (Figure S6), resulting in the elimination of the differences between the two phenotypes. These data show that DNA methylation is dynamic in response to an immune challenge with the parasite; this challenge induces a substantial increase in the 5mC mark and a significant up-regulation of *dnmt2* coupled with a down-regulation of *tet2*. Both these genes have a putative binding sequence of NF-kB in their promoter region, suggesting that they can be induced as a primary response against the challenge. We showed transcriptional differences between phenotypes that may partially explain the susceptibility differences to *Plasmodium*. Furthermore, the rescue of the susceptible phenotype by pharmacological erasure of methylome supports this.

### Inhibition of DNA Methylation Bring *A. albimanus* Mosquitoes Into a Resistance Condition Against *Plasmodium*

**Figure 5:**
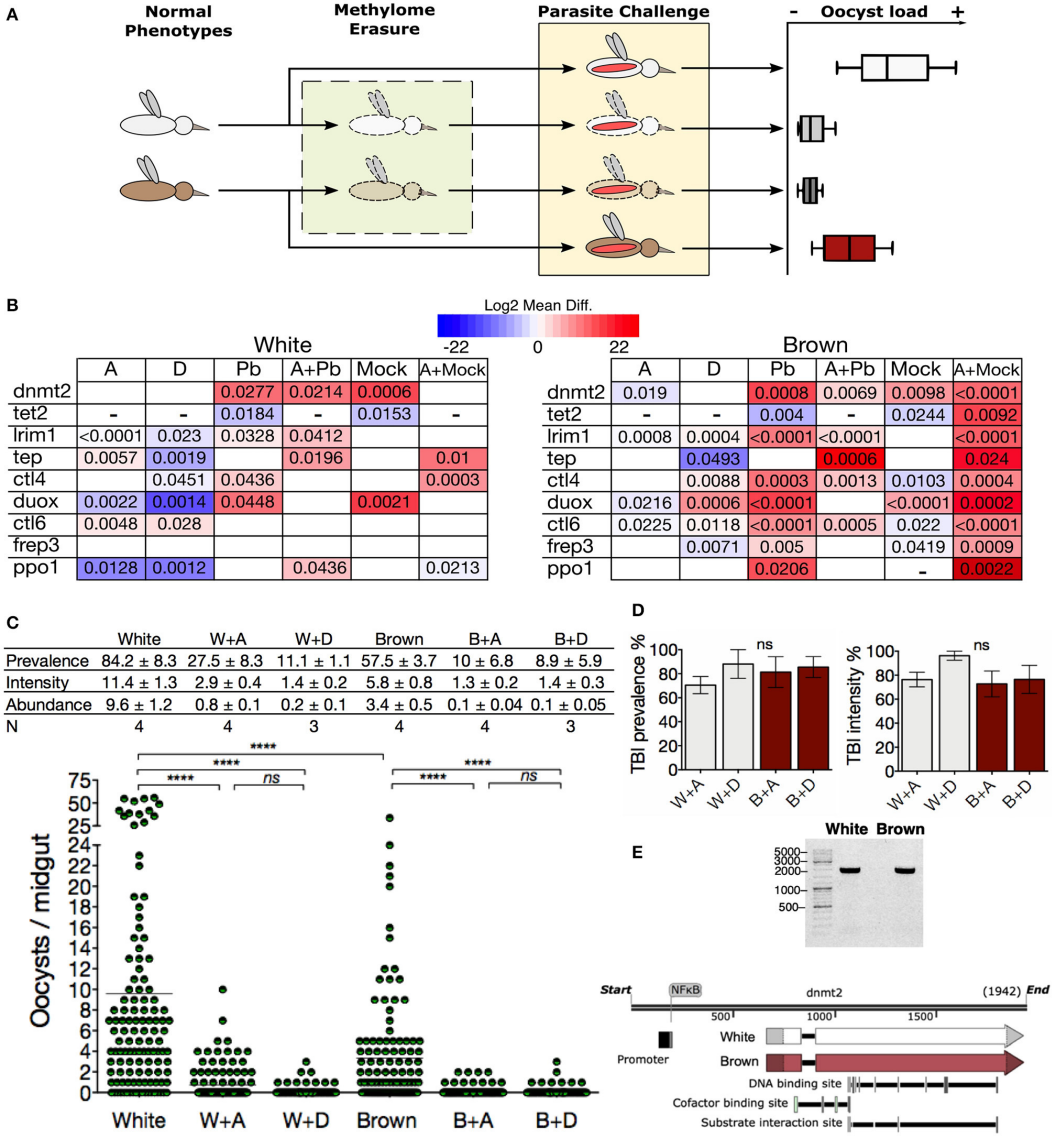
Inhibition of DNA methylation brings *A. albimanus* mosquitoes into a resistance condition against *Plasmodium*. **(A)** Depicts the followed methodology. mRNA samples were taken for each experimental group of mosquitoes (normal phenotypes, methylome erasure, and parasite challenge). **(B)** Midgut gene expression heat map of White and Brown mosquitoes after exposure to azacytidine **(A)**, decitabine **(D)**, and *P. berghei*-infected blood meal (*Pb*). Combination treatments included azacytidine plus infected blood meals (A+*Pb*) and azacytidine plus mock cultures (A+Mock). Color gradient represent mean difference (log_2_) between the basal expression and the treated condition. Only gene expressions with significant differences are shown. A blank box means that no statistically significant change was detected. A dash was placed when no transcript could be detected. Numbers inside the boxes are the *P* values calculated by two-tailed *t* test between each phenotype's control and the treated and/or fed groups. **(C)** Effect of methylation inhibition by azacytidine (A) and decitabine (D) on the infection parameters. Mean ± SEM, *N* = number of experiments with 30 mosquitoes each, analyzed by Kruskal-Wallis and Dunn's multiple comparisons test, ****P < 0.0001. **(D)** TBIs efficacy of the azacytidine (A) or decitabine (D) treatment in White (W) and Brown (B) mosquitoes. Graphs were made with data from **(C)**. **(E)** Long PCR of *dnmt2* for sequencing (Figure S7) and a schematic representation of the *dnmt2* gene.

Subsequently, we examined the impact of the methylation inhibitors on the transcription of immune genes in challenged mosquitoes. We treated the mosquitoes with azacytidine and then challenged them with ookinetes to evaluated transcriptional response to parasite invasion in a state where the epigenetic mark was globally eliminated (Figure 5A). The epigenetically naive state induced by the inhibitor led both phenotypes to respond similarly to the parasite challenge by producing *dnmt2, lrim1*, and *tep*, thus abolishing most of the differences between phenotypes (Figure 5B; A+Pb). TEP1 and LRIM1 are considered the main factors controlling parasite loads in mosquitoes. Their function is similar to the complement system in mammals promoting parasite lysis and melanization (41, 42, 44). In both *Plasmodium*-challenged phenotypes, methylation inhibition increased *tep* transcription (Figure 5B; Pb vs. A+Pb). The increase in *lrim1* transcription, however, was greater in White mosquitoes to that observed in Brown mosquitoes.

On the other hand, the transcription of the infection agonist *ctl4* diminished in both challenged phenotypes when treated with azacytidine. Furthermore, some transcriptional differences persisted; in particular, *ppo1* was also expressed in the White phenotype, while *ctl4* and *ctl6* were only expressed in the Brown phenotype (Figure 5B; A+Pb). The fact that the inhibitors reduced the demethylase *tet2* transcription to undetectable levels, probably contributes to the preservation of the 5mC incipiently placed in response to the parasite challenge. An explanation for these observations is that the epigenetic inheritance of the phenotypes was erased with the inhibitory treatments, so when the mosquitoes encounter the parasite, they started by producing what is probably relevant against the infection. In summary, the response involved the activation of the DNA methylation system, the increment of the 5mC mark, the transcription of anti-*Plasmodium* effectors, and higher resistance to the parasite (Figure 5C). This effect was more noticeable with decitabine, especially in the White mosquitoes.

The transit of the malaria parasite through the mosquito midgut results in a bottleneck that often reduces the parasite population to a single-digit number (38). Hence, transmission-blocking interventions (TBIs) ought to be more effective at this stage of infection. Treatment of mosquitoes with the azanucleosides herein constituted an efficient TBI, with effectiveness ranging between 70 and 96% (Figure 5D), suggesting that DNA methylation could modulate the vector competence.

The *dnmt2* gene was sequenced in an attempt to elucidate the reasons for the observed differences in methylation, but both phenotypes showed identical gene sequences (Figure 5E and Figure S7). The distinct response to methylation inhibition by the azanucleosides between White and Brown mosquitoes, suggests that DNA methylation signaling is divergent in the two phenotypes. Despite this, downstream effects were the same, leveling 5mC and parasite loads between the phenotypes.

## Discussion

This study shows that DNA methylation has an essential role in *A. albimanus* biology. Larvae in which the methylation has been inhibited were unable to grow, and their development was hampered, resulting in early death. This result revealed that in *A. albimanus*, the DNA methylation mechanism is functional and works similarly as in other better-studied insects. In adult mosquitoes, the inhibition of DNA methylation did cause neither death nor other apparent effects besides the modifications in the transcriptional responses. This inhibition translated into both phenotypes becoming considerably more capable of controlling infection loads; however, the mechanism(s) by which the parasite is eliminated are not fully understood. Possible candidates are LRIM1 and TEP1, since both of these transcripts were up-regulated similarly between the phenotypes (Figure 5A).

Interestingly, the azacytidine treatment prevented the transcription of *duox* after a parasite challenge in the two phenotypes. Besides its antimicrobial activity through hydrogen peroxide production, DUOX is associated with mechanisms that reduce damage to the host's microbiota after a blood meal by lowering the intensity of the immune response (47). It has been shown that the silencing of DUOX promotes effective immune responses that include the increment of TEP1, leading to lower infection loads (48). In the particular case of *A. albimanus*, phenoloxidase activity (and *ppo1* gene transcription) is not likely to be related to parasite death. Despite the increment of *ppo1* upon infection in Brown mosquitoes, when the mosquitoes were treated with azacytidine and then challenged, *ppo1* only increased in the White mosquitoes and yet both phenotypes effectively reduced the infection load. The fact that we do not observe parasite remainders or melanization suggests that the parasite is being eliminated by lysis.

The methylation inhibitors removed higher amounts of 5mC in Brown compared to White, though without reaching a total removal of the epigenetic mark. Decitabine or azacytidine act when they are incorporated into DNA (and RNA for azacytidine) during replication, DNA repair, transcription, or demethylation events that involve nucleotide replacement. Then, the DNMT2 interacts with these cytidine analogs and gets irreversibly covalently bonded to the inhibitor during the catalytic reaction. This observation implies that the inhibitors can only inhibit methylation in places where they are incorporated and where the DNMT2 can access them (49), correlating with the higher transcriptional activity of Brown, in which more mark was eliminated. Whereas, in White, with lower transcriptional activity, less 5mC was eliminated. The inaccessibility of certain low activity-genomic regions to the inhibitors or the DNMT2 may also explain why in both phenotypes the 5mC inhibition reached similar levels without lowering any further.

In general, DNA methylation is considered to have gene repression functionalities, primarily when it is located in the promoter regions. However, when located within the gene body it seems to be associated with gene expression (57). In insects DNA methylation is almost exclusively present in gene bodies (3), which explain why the Brown phenotype, with two times more 5mC content, has an overall higher transcription than the White phenotype. After feeding with or without ookinete-containing blood, DNA methylation increases in both phenotypes, although slightly more in the Brown phenotype (**Figure 4A**). This result correlates with the higher transcription of *dnmt2*, and lower transcription of *tet2*. Furthermore, this is in line with the higher transcription of anti-*Plasmodium* genes observed after feeding. The reduction of 5mC after the treatment with the methylation inhibitors lowered the transcription of the immune genes in both phenotypes (Figure 5A). By looking at Figure 5B it can be concluded that the inhibitor treatment lowered the overall transcription but increased the transcription of *lrim1* and *tep*, causing the mosquitoes to respond less intensely but with higher specificity. Despite the above, some transcriptional differences persisted in the phenotypes after the inhibitor treatments which led us to consider as a first possibility the genetic differences. We searched for differences in the *dnmt2* gene; however, it resulted in being identical in both phenotypes after sequencing. A second possibility could rely on the NF-kB family, since all of the genes evaluated in this study have putative binding sites for this family of transcription factors, including *dnmt2* and *tet2*. The exception being *frep3*, which does not have a putative binding site and did not show significant transcriptional changes, at least in White (Table S1). A third possibility is the regulation of other epigenetic mechanisms. It is known that, in many organisms, gene regulation by DNA methylation depends on the context of the surrounding genes (8, 58, 59) and more critical in the context of other epigenetic marks, like histone acetylation and nucleosomal organization (58, 60–62). In this context, the epigenetic state as a whole is a combination of epigenetic modifications. There is a close association between DNA methylation and the modifications in histones (58, 63), in particular, the deacetylation. The methyl CpG binding protein 2 (MeCP2) and other MBD proteins recruit histone deacetylases, which create a dominant repressive chromatin environment in response to changes in the DNA methylation status (64). Considering the lack of knowledge and the complexity of the epigenetic systems, a straightforward relationship between DNA methylation and a particular transcriptional response is not to be forcefully expected, even more knowing that the different epigenetic mechanisms are intertwined. In this regard, the genome of *A. albimanus* has several genes of histone deacetylases among other epigenetic elements that should be taken into account (Table S2).

This report shows a general view of the role of the DNA methylation system in mosquitoes and adds extra information in the changes in vector competence within a mosquito species. Potential directions for future work are the location of 5mC within the different regions that comprise a gene, the regulation of the epigenetic mark by *tet2* and *mbd*, and the connection between the *dnmt2* and the one-carbon metabolism that provides methyl groups for all methylation reactions. Signaling pathways of DNA methylation and epigenetic chromatin circuits are essential aspects that need clarification to have a more comprehensive understanding of this phenomenon. Also, considering possible interactions with other epigenetic mechanisms could clear the view about the epigenetic regulation of immunity, mainly because it is not yet clear how *A. albimanus* gets rid of the malaria parasite.

In this work, we used the interaction of *A. albimanus* and *P. berghei* as a study model to characterize the phenotypic properties of susceptible and resistance phenotypes, making the first analysis of the relationship between DNA methylation, the expression of immune genes, and the susceptible-resistant state of the mosquitoes. We found that the expression profile of anti-*Plasmodium* responsive genes in the midgut is modulated by DNA methylation. This modulation is different between the phenotypes and, regardless of this; the outcome in terms of parasite elimination is the same in both phenotypes upon the erasure of the epigenetic mark, resulting in the elimination of the differences between phenotypes. We found that the 5mC is required for proper larval development but is not necessary for adult survival. In the adult, however, we found that the transcription of *dnmt2* and *tet2* is regulated through feeding and parasite challenges, resulting in an increase of the 5mC mark in the genome. These results render the methylation system in this mosquito as a responsive element to environmental cues, which is further supported by the presence of NF-kB putative binding sites in the promoter regions of these genes.

We have focused on the role of *dnmt2*, the methylating agent, as the factor regulating immunity; however aspects like epigenetic-by-genetic and gene-by-environment interactions are important components to vector competence that should be considered and understood. Here we have unveiled the participation of the methylation system in the malaria mosquito infection, creating new paths to interrupt the transmission of what is the most devastating vector-borne disease.

## Materials and Methods

### Bioinformatics Identification of the Methylation System Genes

The genes involved in the methylation system were identified in the *A. albimanus* genome databank (http://www.ncbi.nlm.nih.gov/genome/11556) and in the vector database (https://www.vectorbase.org/) using the genes of the methylation system of *Drosophila, Mus musculus* and human as the entry query. Sequence analysis and protein domain determination were performed using InterPro (https://www.ebi.ac.uk/interpro/). Promoter analysis was made in (http://www.fruitfly.org/seq_tools/promoter.html) and the prediction of transcription biding sites was obtained in the program AliBaba2 at (http://gene-regulation.com).

### *Anopheles albimanus* Breeding Conditions and Phenotype Selection

The parental *A. albimanus* Tapachula strain and the derived Brown and White phenotypes were reared as described (65). The insectary was maintained at 28–30°C and 70–80% relative humidity, on a 12 h photoperiod. Larvae were kept at a density of 200 individuals per tray with 2 cm deep tap water and fed twice a day with dry ground cat food. The White and Brown phenotypes were selected and separated at the pupal stage based on the presence or absence of a white stripe in the dorsal side. Intermediate phenotypes having a blurry white stripe or green body color were discarded. The phenotypes were kept separated for 20 generations during the course of the experiments. Reoccurring phenotypes were systematically depurated each generation.

For determining the developmental stage of the larvae, the moltings were traced by the presence of exuviae, and confirmed by comparing the exuvial head capsules size to the larval head size. Measurements were made in a stereoscopic microscope with an ocular micrometer. Adult mosquitoes were maintained under the aforementioned insectary conditions at a density of 200 mosquitoes per 5 liter container. Feeding was carried out *ad libitum* with 8% w/v sucrose in ddH_2_O, delivered on sterile cotton pads and changed every 24 h. For breeding, mosquitoes were fed rabbit blood at 37°C though a feeding membrane. Oviposition cups were provided 48 h after feeding. The eggs were collected after 24 h and allowed to hatch before transferring to the plastic trays.

### Ookinete Culture and Mosquito Infections

*Plasmodium berghei* ANKA strain (kindly donated by Dr. R. Sinden, Imperial College London), constitutively expressing the green fluorescent protein, was used throughout the experiments. Ookinetes were cultured as described (66). Briefly, male BALB/c mice from 6 to 8 weeks of age were intraperitoneally treated with phenylhydrazine (6 mg/ml in 0.8% saline) 2 days before the inoculation of 2–4 × 10^8^ parasites via the same route. When the parasitemia reached 15–25%, and after verifying gametocyte viability, the infected blood was extracted by cardiac puncture with a heparinised syringe from the CO_2_ euthanized mice. To allow for ookinete formation, the blood was then incubated at 19–20°C for 20–24 h in ookinete medium (1:4, blood:medium), which consists of RPMI-1640 at pH 8.3 supplemented with 23.81 mM sodium bicarbonate, 0.37 mM hypoxanthine, 25 mM HEPES, PSN (0.05 mg/ml penicillin, 0.05 mg/ml streptomycin and 0.1 mg/ml neomycin; Gibco) and 20% heat inactivated fetal bovine serum (FBS).

Mosquitoes were infected with ookinetes by the standard membrane alimentation methodology. Only cultures containing > 7 exflagellation centers per 400X field of view were used. The ookinetes were counted in a Neubauer chamber and the culture was centrifuged at 2,000 rpm for 5 min. The ookinete pellet was resuspended to a final concentration of 400 ookinetes per microliter of FBS for the feedings. As control, mock cultures were prepared with the blood from non-infected mice. Mosquitoes of 5 days post-emergency (dpe) were starved for 6 h and allowed to feed for 30–60 min from the membrane glass feeder heated at 37°C. Only fully engorged females were kept for further experimentation at 19–20°C and 70–80% relative humidity, on a 12 h photoperiod. Infected and mock-infected mosquitoes fed on cotton pads moistened with 8% sucrose solution with 0.05% PABA. Appraisal of the infection was measured 3 days post infection (dpi) by dissecting in PBS the midguts of 30 cold anesthetized females and observing the GFP-expressing oocysts by fluorescence microscopy. The mosquitoes were used for the assays only if the infection prevalence was 80% or higher in the susceptible-White phenotype control group.

Transmission blocking efficiency was calculated as in (67).

### Nucleic Acids Methylation Inhibition Assays

For inhibiting the methylation of nucleic acids, the DNA/RNA methylation inhibitor azacytidine (5-azacytidine), and the DNA exclusive methylation inhibitor decitabine (5-Aza-2′-deoxycytidine; both purchased from Sigma-Aldrich) were used. Larvae and mosquitoes were maintained under experimental conditions optimal for *P. berghei* (see above). To evaluate the impact of nucleic acid methylation during development, 100 larvae of 1 h post hatching were treated with 50 μM azacytidine in 6 well-culture plates. To avoid compound accumulation, the treatment was replenished every 48 h by moving the larvae to an adjacent well-containing fresh treatment. Every 24 h larval survival was monitored and growth was measured taking the cephalic-caudal length. Developmental stages were assessed by collection of exuviae and dead larvae were removed.

Methylation inhibition in adults started at 2 dpe by *ad libitum* feeding on cotton pads moistened with 50 μM of azacytidine or decitabine in a solution of 8% sucrose, 0.05% PABA and PSN. The treatments lasted 72 h, replacing the cotton pads every 24 h. In the case of assays requiring infected mosquitoes, these were infected after the inhibition treatments were concluded. Dissection of midguts destined for RNA or DNA extractions, was carried out at 5 dpe (after 72 h of treatment with inhibitors) and also at 6 dpe (24 h post infection). Midguts were dissected as described above, thoroughly washed and stored at −20°C. For infected mosquitoes, the midguts were used for RNA or DNA extraction only if the infection was confirmed at 3 dpi.

### Effect of Azacytidine on *P. berghei* Survival and Development

To determine whether azacytidine affects parasite survival or development, the ookinetes were purified and then cultured in the presence of 50 μM azacytidine. Mock treated (PBS) and 1 mM H_2_O_2_ were used as controls. The ookinetes were cultured to allow oocyst formation at 19–20°C in oocyst medium consisting of Schneider's insect medium at pH 6.8 supplemented with 23.8 mM sodium bicarbonate, 3.68 mM hypoxanthine, PSN, 44 μM PABA, 0.2% lipids/cholesterol (Gibco) and 15% FBS (68). Parasite survival and development were determined with fluorescence microscopy at 0, 12, 36 and 72 h of culture.

### gDNA and RNA Extraction

For gDNA extraction, the midguts of 30 mosquitoes per treatment were dissected and incubated overnight at 55°C in lysis solution and treated with proteinase K and RNase A. RNA was extracted from 30 midguts per sample by employing the TRIzol (Thermo Fisher Scientific) protocol. DNA and RNA samples were stored at −20°C until further use. For cDNA synthesis, RNA extracts (1 μg) were treated with DNase I and 500 ng/μl of OligodT were added. After 10 min at 70°C, the master mix (1 mM dNTP's, 0.01 mM DTT, 20 units of RNase inhibitor and reverse transcriptase buffer) and 200 U/μl M-MLVRT were added.

### PCRs

*dnmt2, tet2*, and *s7* genes were amplified using 10 ng cDNA, 0.5 μM for each primer (Table S3), 1 μM dNTP's, 3.25 mM MgCl_2_, 1X DreamTaq Green buffer (with 2 mM MgCl_2_) and 1.25 U DreamTaq Green DNA pol (Thermo Fisher Scientific). *dnmt2* long PCR, including the 5′ and 3′ UTR and promoter regions, was amplified from midgut DNA. The reaction mix contained 100 ng of gDNA, 0.5 μM of each primer (Table S4), 1 μM dNTP's, 2.5 mM MgCl_2_, Long PCR 1X buffer (with 1.5 mM MgCl_2_) and 2 U of Long PCR enzyme (Thermo Fisher Scientific). PCR reactions were carried out in a T100™ (BIO-RAD) thermocycler and visualized on agarose gel electrophoresis. The PCR band was purified with the GeneJET Gel Extraction kit (Thermo Fisher Scientific). The PCR product was sequenced at the Unidad de Síntesis y Secuenciación de ADN of the Instituto de Biotecnología-UNAM.

### RT-qPCR

*dnmt2, tet2, ctl4, ctl6, lrim1, tep, duox, ppo1, frep3*, and *s7* genes were amplified as following: a mixture of 5 ng cDNA, 0.25 μM oligo (Table S3) and Master Mix SYBR Green 1X was loaded on 96 well-plates on a ViiA™ 7, Applied Biosystems thermocycler. Amplification efficiency was determined with LinRegPCR. Data were normalized to S7 gene amplification.

### gDNA Digestion Sensitive to Methylation

Midgut gDNA (1 μg) was digested with 1 unit of *HpaII* (Thermo Fisher Scientific) in 1X Fast Digest buffer for 1 h at 37°C. gDNA digestion products were visualized on agarose gel electrophoresis.

### Dot Blot

Midgut gDNA of 5 dpe female mosquitoes was spotted on a nitrocellulose membrane (Hybond^®^ ECL™), dried and fixed with UV light. An anti-5^m^C antibody (Zymo research, A3001-200) was used in conjunction with αPRP (1:1,000, GERPN2108-ECL™ Western Blotting Analysis System). Fluorescence was developed on a KODAK BioMaxLight film. The films were scanned and a densitometric analysis was performed using ImageJ to calculate the mean pixel intensity of the dots.

### High Performance Liquid Chromatography (HPLC)

Methylation groups in DNA and RNA were quantified by HPLC by the following methodology (69). Briefly, gDNA was digested with DNase I, S1 nuclease and treated with alkaline phosphatase. For RNA digestion, RNase H and P1 nuclease were used following the same methodology as for gDNA. The nucleosides were derivatized with bromoacetophenone. HPLC analysis was performed on an Agilent Series 1100 utilizing an Agilent Sorbax C18 (250 × 4.6 mm, 5 μm) column and a Supelco pre-column. Derivatized nucleosides were fluorometrically detected at excitation/emission wave lengths of 306/378 nm.

### PO Activity

PO activity was measured as described (70). Pools of 30 mosquitoes were macerated and centrifuged at 10,000 g for 10 min at 4°C. PPO was activated with isopropanol to quantify the total enzyme. PO was inhibited with phenylthiourea and PBS was used to measure basal enzyme activity. L-DOPA was used as the substrate for PO, which is transformed into the dye dopachrome. Auto-oxidation controls (L-DOPA only) and blanks (macerated mosquitoes) were included. PO activity was measured every minute for 30 min at 490 nm in a microplate reader (ELISA iMark, BIO-RAD).

### NO Quantification

Nitrites and nitrates were evaluated by the Griess assay (71). Pools of 30 mosquitoes per treatment were macerated and centrifuged twice at 10,000 g for 10 min at 4°C. Proteins were eliminated with ZnSO_4_. Nitrates were reduced into nitrites using VCl_3_ immediately followed by the addition of sulfanilamide and NED. The reaction was incubated for 15 min at RT and the absorbance was measured at 490 and 630 nm in a microplate reader.

### Statistical Analysis

Data was analyzed in Prism 6 statistical software. The sample size for mosquito infections and nucleic acids extraction was determined according to Churcher et al. (67). Data were tested for normality by the D'Agostino and Pearson omnibus normality test. Survival of larvae, adults and parasites was analyzed using Mantel-Cox test (Log-rank test). Larval growth was analyzed with Kruskal-Wallis and Dunns multiple comparisons test. The infection parameters were analyzed through Mann-Whitney or Kruskal-Wallis and Dunn's multiple comparisons test. PCR, HPLC, PO, NO and TBIs results were analyzed by conducting a two-tailed *t* test or ANOVA followed by a Tukey's multiple comparison test.

## Data Availability Statement

The raw data supporting the conclusions of this manuscript will be made available by the authors, without undue reservation, to any qualified researcher.

## Ethics Statement

The Ethics, Biosafety, and Research Committees of our institution evaluated and approved this project. The handling of animals and the experimentation with mice and mosquitoes was carried out adhering to the criteria of the aforementioned committees.

## Author Contributions

FC-P conceived the project, designed and performed most of the experiments, analyzed the data and wrote the original draft. JH-T assisted in experimentation and data collection. BR-T produced the parasites. GH-S performed the HPLC experiments. HL-M supervised the project and contributed in experiment design. BR-T, RC, and FC-P interpreted the data and wrote the final version of the manuscript.

### Conflict of Interest

The authors declare that the research was conducted in the absence of any commercial or financial relationships that could be construed as a potential conflict of interest.
