# Supplementary material for: DNA Methylation in Anopheles albimanus Modulates the Midgut Immune Response Against Plasmodium berghei
Source: Front Immunol. 2020 Jan 14;10:3025. doi: 10.3389/fimmu.2019.03025 (PMC6970940; doi:10.3389/fimmu.2019.03025)
Supplement: Supplementary file 1 [file Data_Sheet_1.DOCX]

Supplementary Material

## Supplementary Figures


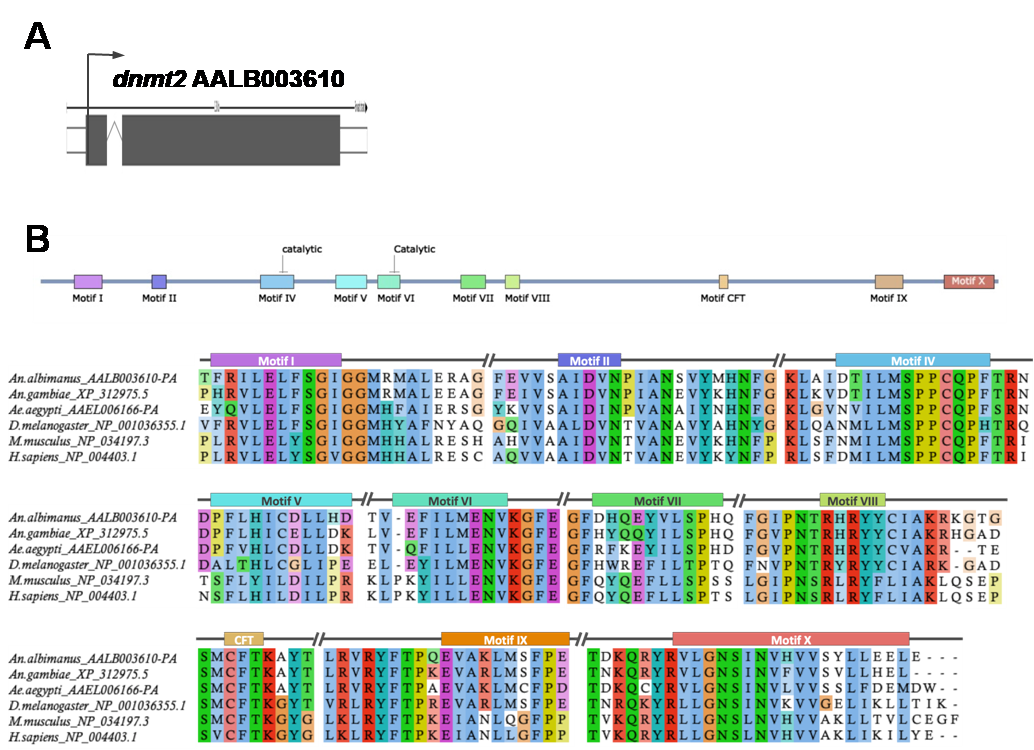


Supplementary Figure. 1. (A) Gene schematic of *dnmt2*. The gene is located in the SuperContig KB672468 from base 4,759,720 to 4,761,021. It is constituted by two exons forming a final transcript of 1,236 base pairs (bp) encoding for a 344 amino-acid (aa) protein. (B) Protein schematic (above) and multiple sequence alignment (below) of the DNA methyltransferase 2 (DNMT2) protein motifs between several species. The C-terminal C-5 cytosine methyltransferase catalytic domain (IPR001525) is shared by motifs IV and VI. Amino acids are color coded by their properties following ClustalW guidelines. Accession numbers are provided after the species name.


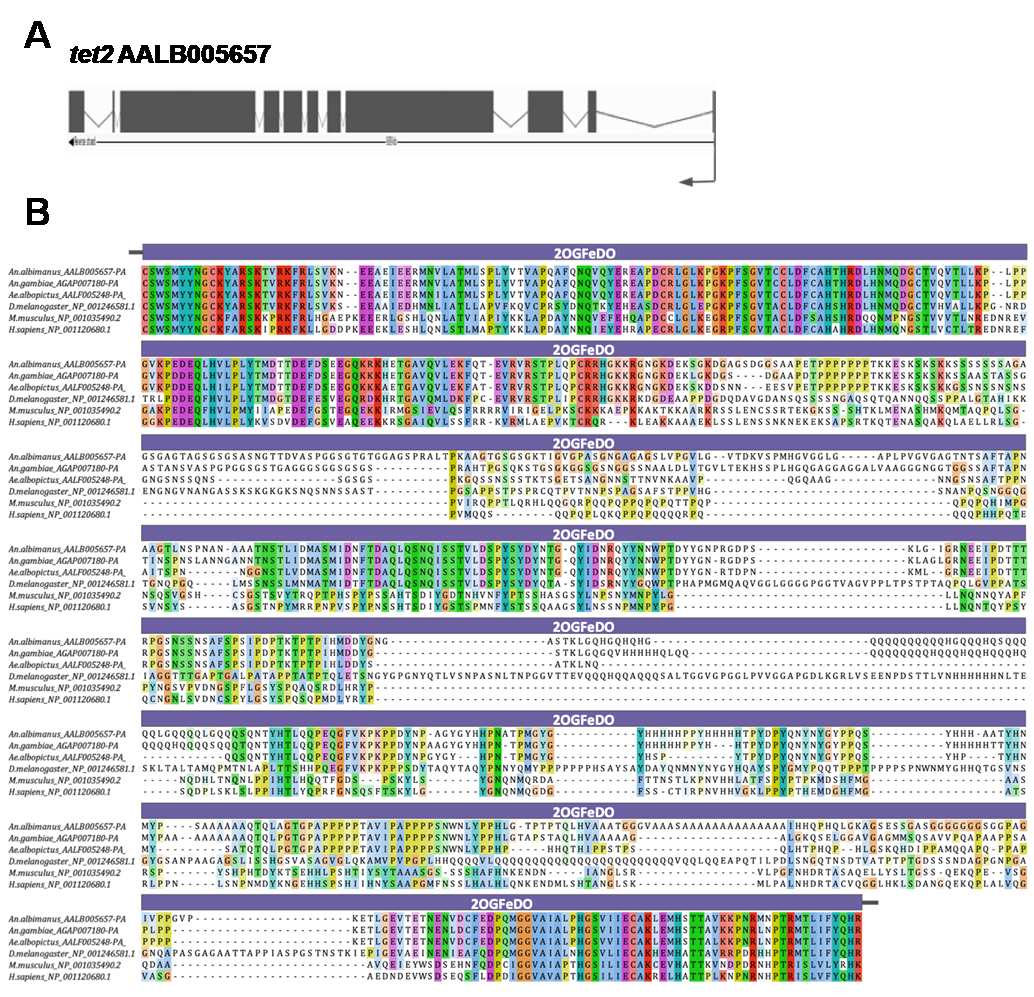


Supplementary Figure 2. (A) Gene schematic of *tet2*. The gene is located in the SuperContig KB672286 from base 22,682,404 to 22,692,296. It consists of 11 exons giving a putative mRNA of 6,153 bp that encode for a 2,050 aa protein. (B) Multiple sequence alignment of the canonical Ten-eleven Translocation 2 (TET2) 2-oxoglutarate- Fe-dependent dioxygenase catalytic motif (IPR024779) between several species. Amino acids are color coded by their properties according to ClustalW guidelines. Accession numbers are provided after the species name.


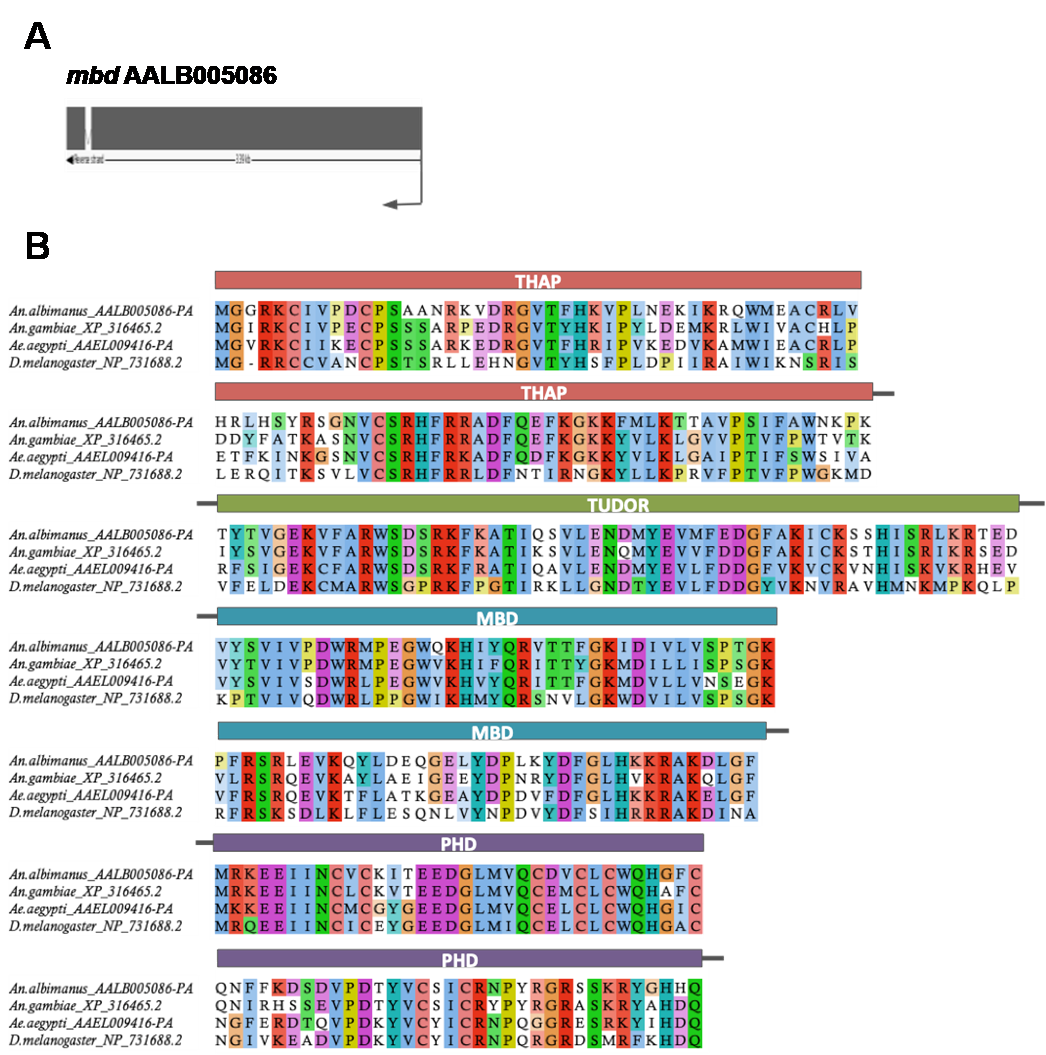


**Supplementary Figure 3. (A)** Gene schematic of *mbd*. The gene is located in the SuperContig KB672286 from base 16,080,137 to 16,083,522. It contains 2 exons that generate a 3,324 bp mRNA encoding for a 1,107 aa protein. **(B)** Multiple sequence alignment of the methyl cytosine binding protein (MBD) motifs of several insect species. The predicted sequence contains a methyl-CpG DNA specific binding motif from the methyl-CpG DNA binding protein family (IPR001739), as well as the THAP-type (IPR006612) Zinc Finger domain, the PHD-type (IPR001965) Zinc Finger domain and the Tudor (IPR002999) domain. Amino acids are color coded by their properties according to ClustalW guidelines. Accession numbers are provided after the species name.

**
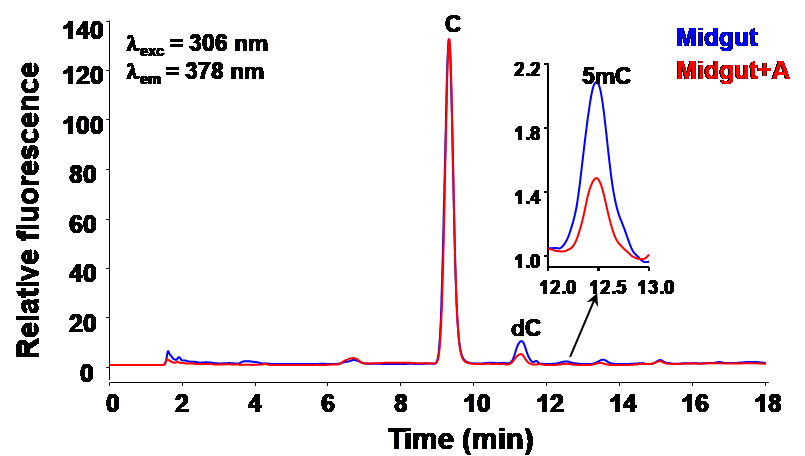
**

**Supplementary Figure 4.** Detection of 5mC in RNA extracts from midguts of azacitidine-treated mosquitoes. cytidine (C), deoxycytidine (dC) and 5-methylcytidine (5mC).

**
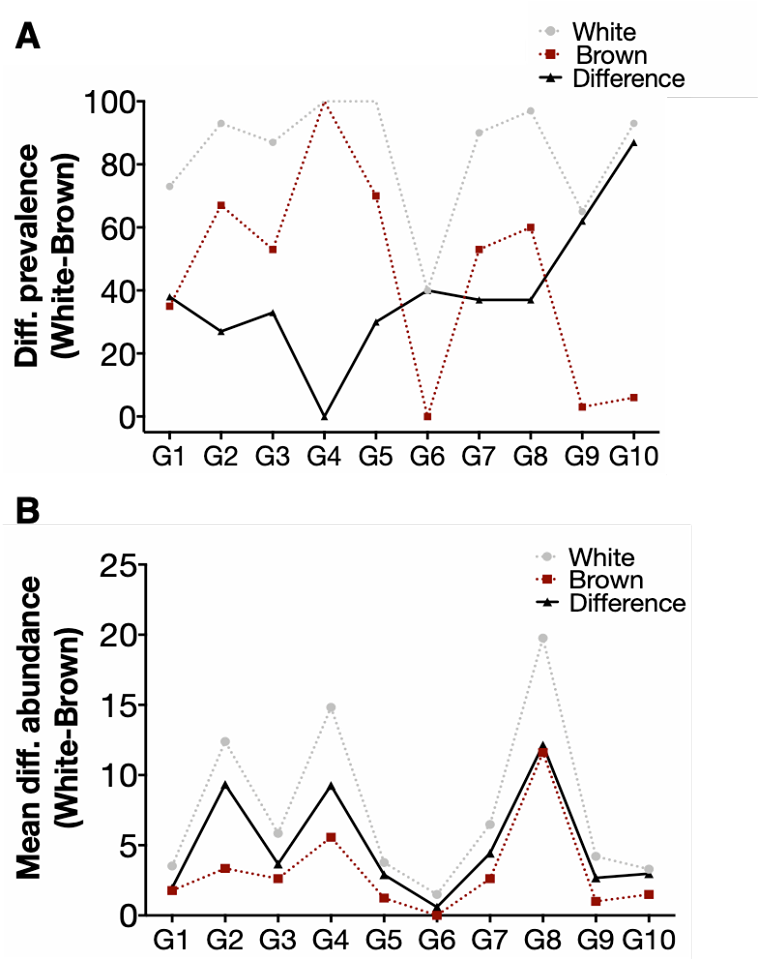
**

**Supplementary Figure 5. Intergenerational *P. berghei* infections.** Difference in infection prevalence **(A)** and abundance **(B)** in 10 generations of the Brown and White phenotypes.


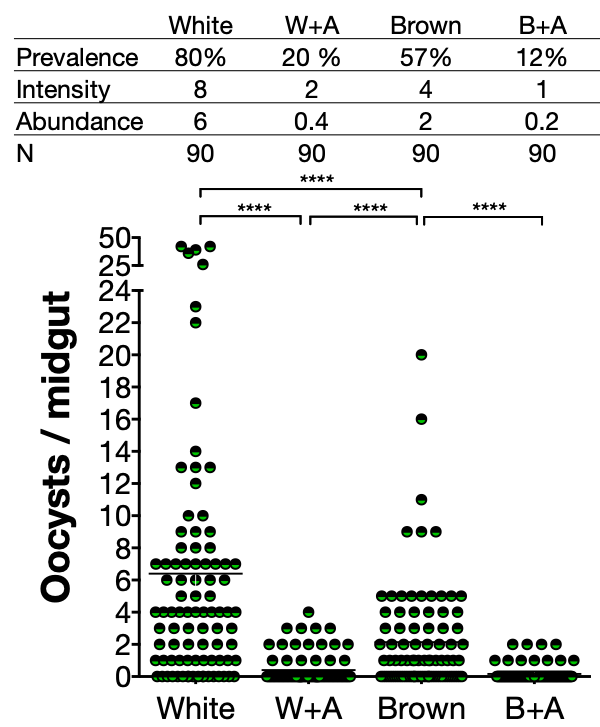


**Supplementary Figure 6.** Effect of methylation inhibition by azacytidine on the infection parameters in both phenotypes. N = number of mosquitoes. (W) White mosquitoes, (B) Brown mosquitoes, (A) azacitidine. Data was analyzed by Kruskal-Wallis and Dunn’s multiple comparisons test, *****P* < 0.0001.

**
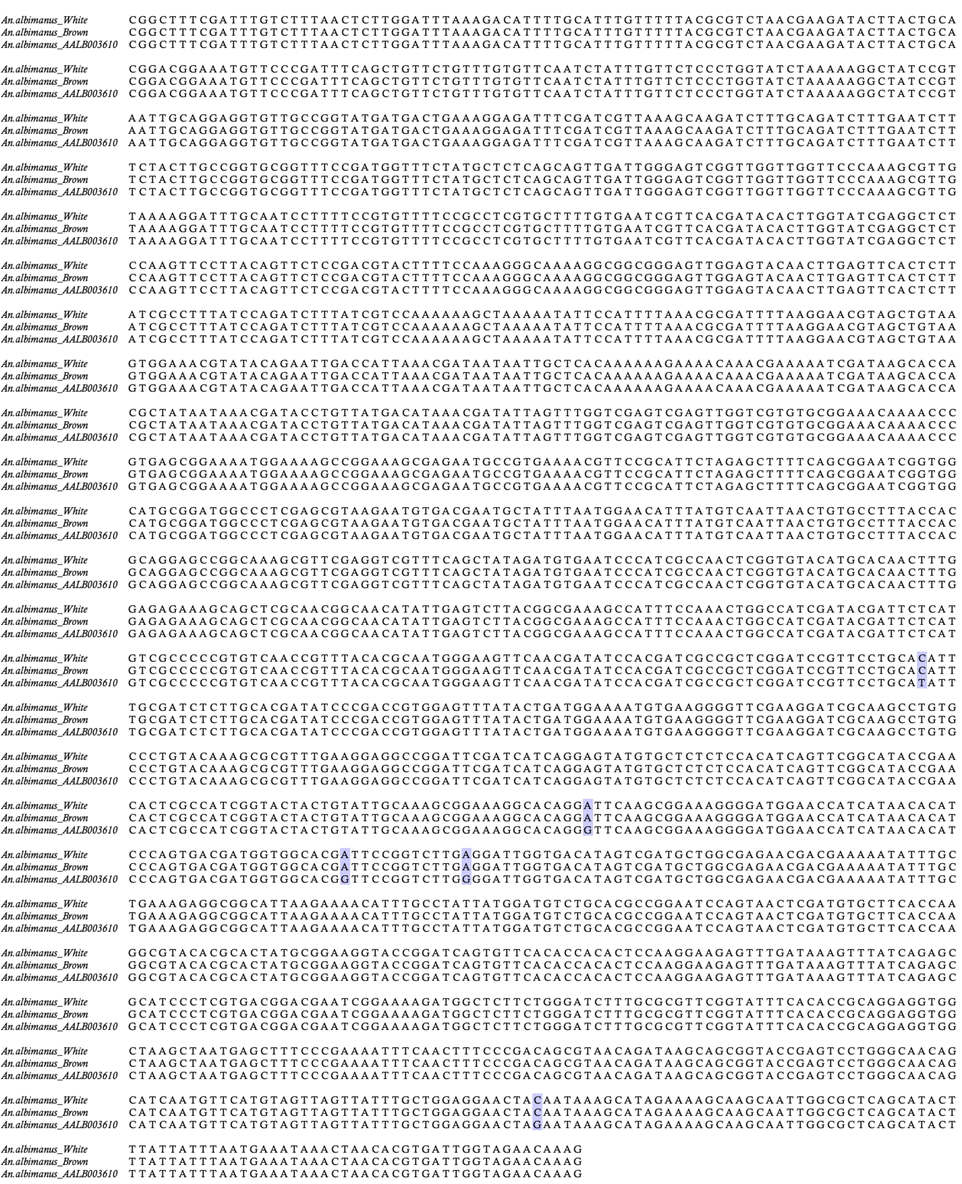
**

**Supplementary Figure 7.** *dnmt2* multiple sequence alignment of the White and Brown phenotypes compared to the reference *A. albimanus* sequence. Colored background denotes ≤ 66 % identity.

**Table S1.** *dnmt2* and *tet2*, as well as several of the genes evaluated are under the control of the NF-kB family of transcription factors.


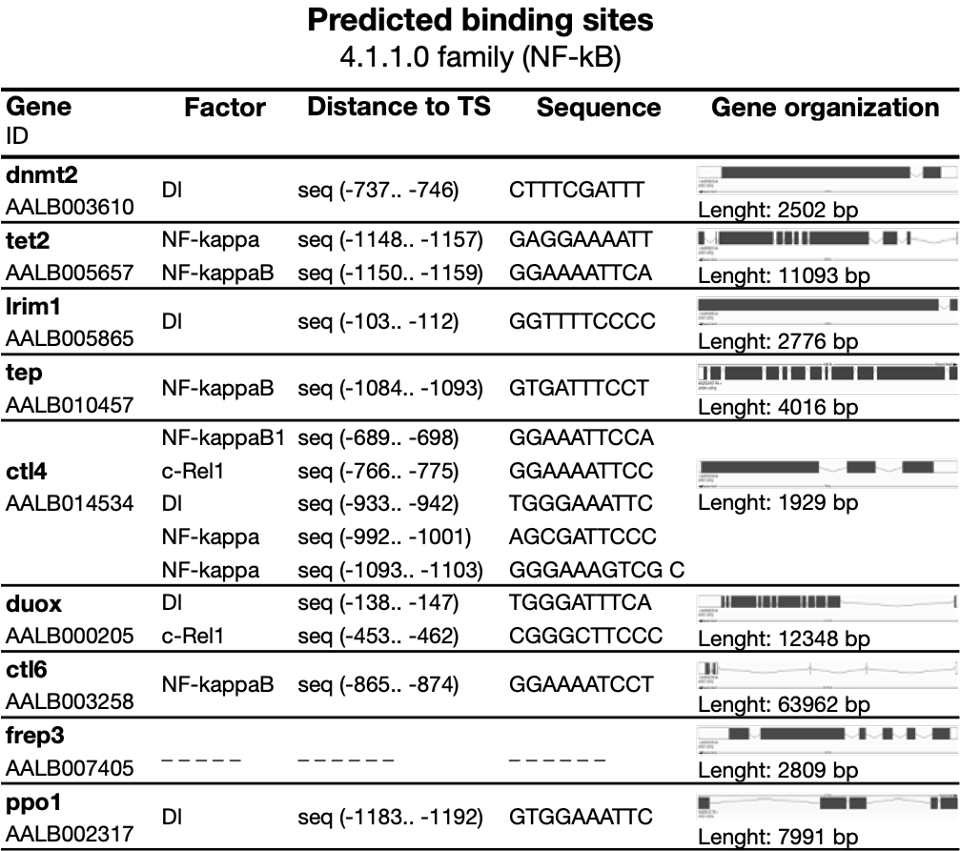


**Table S2.** Number of *Anopheles albimanus* genes that are orthologous to *D. melanogaster* genes involved in epigenesist.


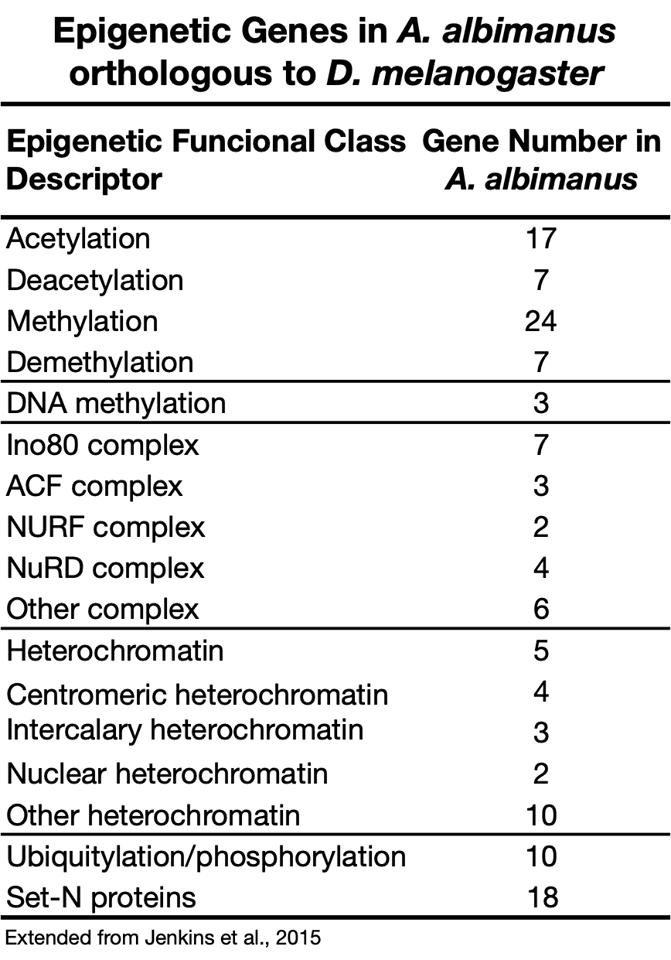


**Table S3.** Oligonucleotide primers used for qPCR amplifications.


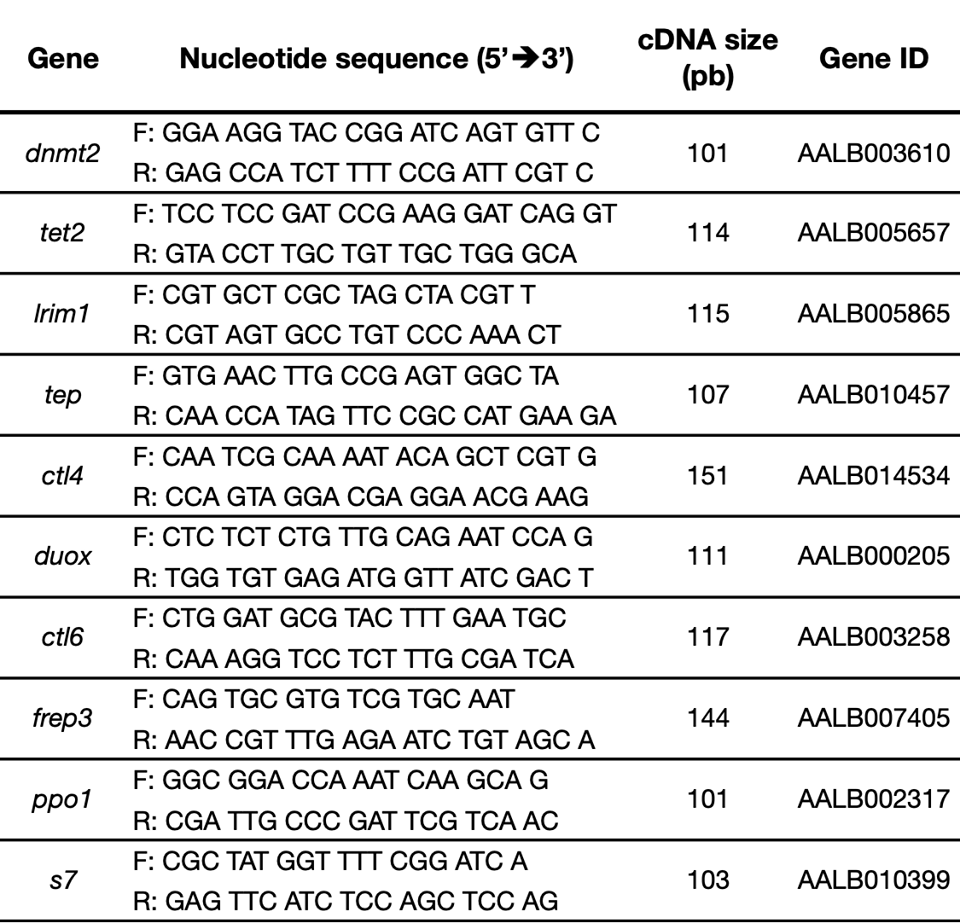


F= forward, R= reverse.

**Table S4**. Oligonucleotide primers used for long PCR and sequencing.


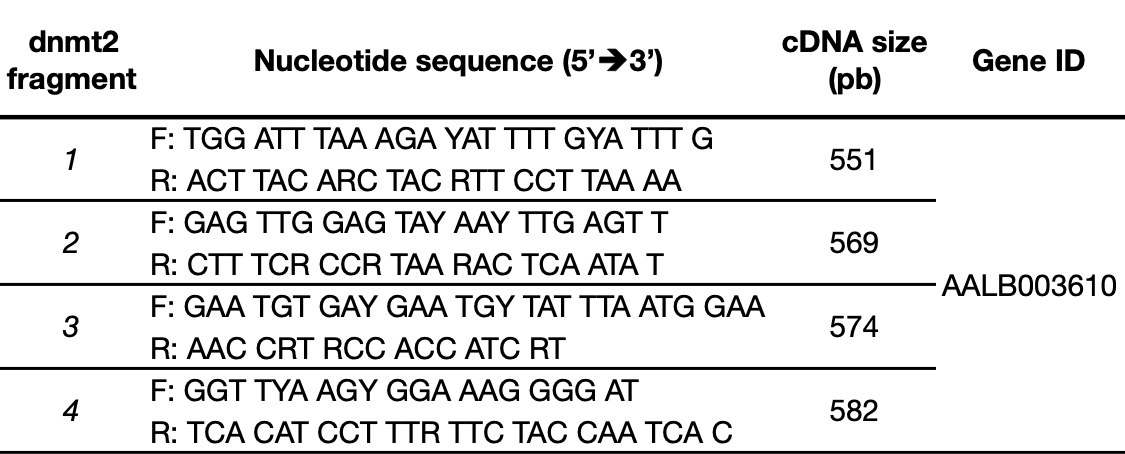


F= forward, R= reverse.
